# Development of laparoscopic skills in Medical students naive to surgical training

**DOI:** 10.1590/S1679-45082014AO3237

**Published:** 2014

**Authors:** Worens Luiz Pereira Cavalini, Christiano Marlo Paggi Claus, Daniellson Dimbarre, Antonio Moris Cury, Eduardo Aimoré Bonin, Marcelo de Paula Loureiro, Paolo Salvalaggio

**Affiliations:** 1 Universidade Positivo, Curitiba, PR, Brazil.; 2 Instituto Jacques Perissat, Curitiba, PR, Brazil.

**Keywords:** Students, medical, Laparoscopy, Models, educational, Surgical procedures, operative/education

## Abstract

**Objective:**

To assess the acquisition of basic laparoscopic skills of Medical students trained on a surgical simulator.

**Methods:**

First- and second-year Medical students participated on a laparoscopic training program on simulators. None of the students had previous classes of surgical technique, exposure to surgical practice nor training prior to the enrollment in to the study. Students´ time were collected before and after the 150-minute training. Skill acquisition was measured comparing time and scores of students and senior instructors of laparoscopic surgery

**Results:**

Sixty-eight students participated of the study, with a mean age of 20.4 years, with a predominance of first-year students (62%). All students improved performance in score and time, after training (p<0,001). Score improvement in the exercises ranged from 294.1 to 823%. Univariate and multivariate analyses identified that second-year Medical students have achieved higher performance after training.

**Conclusions:**

Medical students who had never been exposed to surgical techniques can acquire basic laparoscopic skills after training in simulators. Second-year undergraduates had better performance than first-year students.

## INTRODUCTION

Laparoscopy has made a revolution in surgery in the last decades. Surgeries are now performed without open the abdominal cavity, with quicker and less traumatic recovery. With the creation of this new technique, challenges have emerged in how to train surgeons on the skills necessary for its efficient and safe practice.^(^1^)^ Among the difficulties for the acquisition of skills in laparoscopic surgery are the loss of depth perception and of tactile sensation, the “fulcrum” effect (instruments that move in a point fixed to the abdominal wall causing inverse paradoxical movements), and finally the modifications of the hand-eye coordination.^(^2^)^ In order to solve the problem, the concept of training in simulators, also known as “black boxes”, was created.^(^3^, ^4^)^

Training in simulators is designed to improve and transfer skills acquired in the training laboratory to the operating room.^(^5^,^6^)^ With the intention of establishing a minimum standard of training and skill acquisition, the Society of American Gastrointestinal and Endoscopic Surgeons (SAGES) created an educational program called Fundaments in Laparoscopic Surgery (FLS).^(^7^)^ This program is based in a series of validated exercises, developed over skills peculiar to the practice of laparoscopy.^(^6^,^8^)^ With the use of FLS the acquisition of skills can be measured in a qualitative and objective way, based on efficiency and precision in performing the surgical tasks.^(^6^)^

Besides approaching skills acquisition, it is important to identify how learning occurs.^(^9^)^ Learning is influenced by many complex factors, including the possible innate ability of the surgeon, as well as previous surgical experience.^(^9^)^ The identification of factors that facilitate or hinder the acquisition is key to minimize the learning curve.^(^9^)^

Nevertheless, how learning happens and the possible factors influencing skill acquisition are not known in detail. More specifically, how people with no previous training in laparoscopy acquire and/or develop specific skills, what is the speed and the limits of acquisition or the influences of a systematized training are not fully understood.

## OBJECTIVE

To assess the acquisition of laparoscopic skills by Medical students trained in a simulator.

## METHODS

A prospective, longitudinal study with first and second year Medical students from the *Universidade Positivo*, in Curitiba (PR) was conducted from June 1^st^, 2012 through September 1^st^, 2013.

The volunteer students filled in a demographic questionnaire and signed an Informed Consent authorizing the disclosure of information for the study. The study was approved by the Institutional Review Board of the *Universidade* *Positivo*, under the number 51,598. (CAAE: 05247812.6.0000.0093)

The demographic questionnaire collected data on age, gender, year of graduation, intention of following a surgical career, dominant hand, and manual ability developed by the practice of videogame.

The students were separated in training groups on the simulator box for a total of 150 minutes. There were two training sessions, separated by a one-week interval time.

The volunteer students’ time and score previous to training in simulators were used as control.

The performance in simulators of instructor surgeons from the Minimally Invasive Surgery Service of the *Universidade Positivo* served to form a group of instructors to record time and score. This group had more than 10 years of practice in advanced laparoscopy, and performed at least 500 surgeries, being locally and nationally known by colleagues of the specialty. Time and score of the instructors group were the average of all those senior surgeons. This time and score were also considered as final goals in the skills acquisition by the students included in the study.

Therefore, the students’ time and score were assessed taking into account the time and score of the instructor surgeons.

### Tasks and penalties

The tasks used to measure the acquisition of skills in laparoscopy mimic the ones originally described in the FLS.^(^8^,^10^,^11^)^

### Peg transfer

There was a 12-peg pegboard in the center of the screen. In one side, there were six rings in six pegs. Each of the six objects should be raised using the non-dominant hand, and placed on the other side. After moving the six structures, all should be returned to the initial position.^(^8^)^ The exercise began when the dissectors appeared on the screen and ended with the placement of the last ring. Penalty was applied for each object that fell off the visual field or off-reach, 10 seconds being counted for each error. The time limit was 300 seconds.

### Cutting

In the center of the screen there was a 10x10cm gauze, with a 5cm-diameter circle drawn in the middle The pre-drawn circle should be cut with scissors.^(^8^)^ The exercise began when the gauze was touched and ended when the circle was entirely cut. Penalty was applied in comparison to the circles cut by the experts, with a maximum deviation tolerance of 1mm. Circles that exceeded that would score a penalty for each millimeter. Time limit was 300 seconds.

### Passing

In the center of the screen there was a plate with many rows of shafts with holes. The wire should be guided through the orifices of all the rows, to the end of the shafts. The task started when the guide-wire was suspended and ended when it passed the last hole.^(^10^)^ The time limit was 300 seconds. The penalty was scored for each hole the guide-wire did not go through, counting 10 seconds per penalty.^(^12^)^

### Intracorporeal knot

In the center of the screen, there was a structure with a fixed surgical suture. One double and four single knots should be tied with the needle-holder and against the needle- holder.^(^8^)^ The exercise began when the devices appeared in the monitor and ended when the last knot was tied. The penalty was for loose knots, scoring 10 seconds for looseness.^(^11^,^12^)^ Time limit was 300 seconds.

### Suture

In the center of the screen, there was a Penrose drain with a slit. On each side of the slit there were two marks. The marks should be united by a suture with needle and thread.^(^8^)^ The exercise began when the device was visible on the screen and ended when the suture was complete. Penalty was applied when there was deviation from the marks or the knots were loose. One second was scored for each millimeter away from the demarcation and 10 seconds for loose knots.^(^11^, ^12^)^ Time limit was 600 seconds.

### Penalties and scores

The baseline times and penalties were established according to previously published methodology.^(^5^,^6^,^8^)^ The gross score for each task was the time of accomplishment minus the baseline time, minus the penalties. Therefore, higher scores mean better performance. If the participant was not able to complete the exercise and exceeded the limit time, zero time was assigned, and, as a consequence, the score was zero.^(^13^)^

The score was a percentage of the gross scores in relation to the scores of the experts, according to the formula below:^(^6^,^13^)^


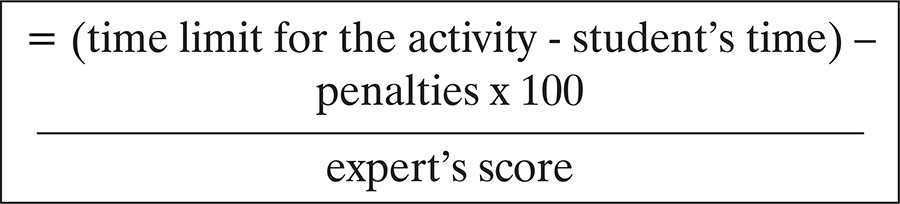


The score for each student was the average score of each task.

The results of the quantitative variables were described as averages, medians, minimum and maximum values, and standard deviations. Qualitative variables were described by frequency and percentage. To compare before and after training evaluations, in relation to quantitative parameters, the Wilcoxon non-parametric test was used. For dichotomic qualitative variables the analysis was done using the McNemar test. When comparing the two groups in regard to age, the Student *t* test for independent samples was applied. To evaluate the association between two dichotomic qualitative variables, the Fisher’s exact test was used. The normality condition of the variables was analyzed by the Kolmogorov-Smirnov test. A univariate analysis was performed to verify values that predict students’ performance gain, defined as final place among the 20 best scores obtained. Multivariate analysis was then performed to identify factors which contributed for this gain individually. The p values <0.05 indicated statistical significance. Data were analyzed using the Statistical Package for the Social Science software (SPSS) v.20.0.

## RESULTS

The study included 68 students with similar gender distribution. There were more 1^st^ year students and with the intention of pursuing a career in the surgical field in the future. Further demographic information is displayed on table 1.

**Table 1:** Demographic data of Medical students evaluated on laparoscopic surgery simulator

Demographic data	
Age (mean)	20.4 (17 ±27)
Standard deviation	1.8
Female n (%)	38 (55)
1st year students, n (%)	42 (62)
Surgical carrier (yes), n (%)	57 (84)
Manual skill (yes), n (%)	37 (54)
Videogame practice (no), n (%)	35 (51)
Dominant hand (right), n (%)	63 (93)

**Figure 1:**
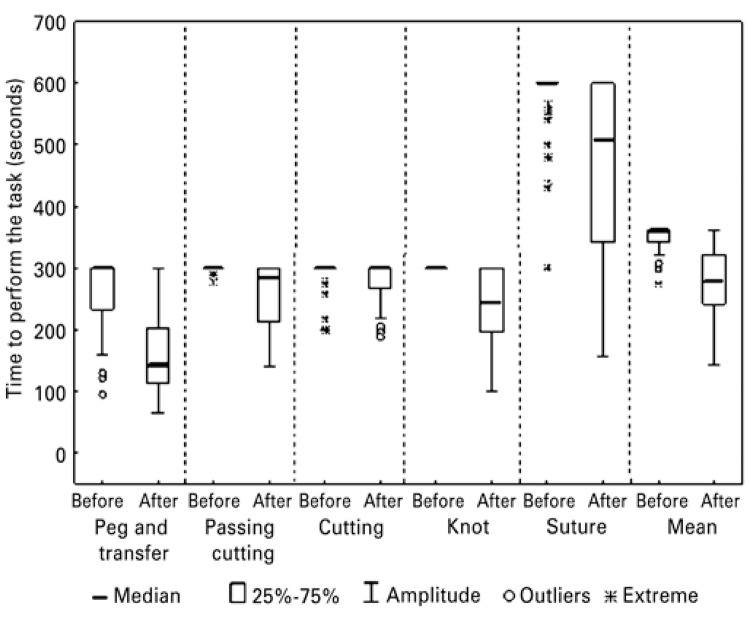
Time to perform exercises by Medical students, before and after training in a laparoscopic surgery simulator

The times obtained were analyzed verifying a possible improvement after training. In figure 1, times in seconds for performing each exercise, measured before and after training, improved for all exercises. The comparison between the two training steps was statistically significant (p<0.001).

**Figure 2:**
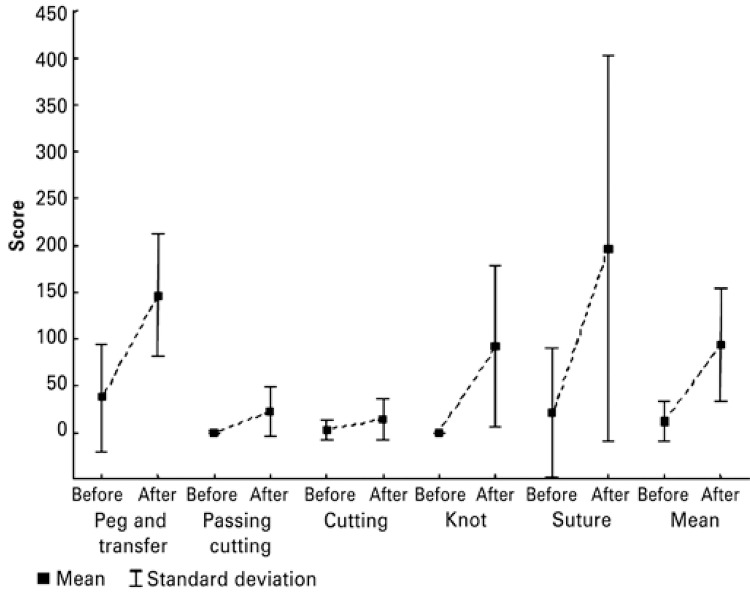
Performance before and after training for different laparoscopic exercises by Medical students

The conversion of time into scores is depicted on figure 2. There was a variation in improvement from 294.1 to 823%, depending on the exercise. Every score had a p<0.001.

**Figure 3:**
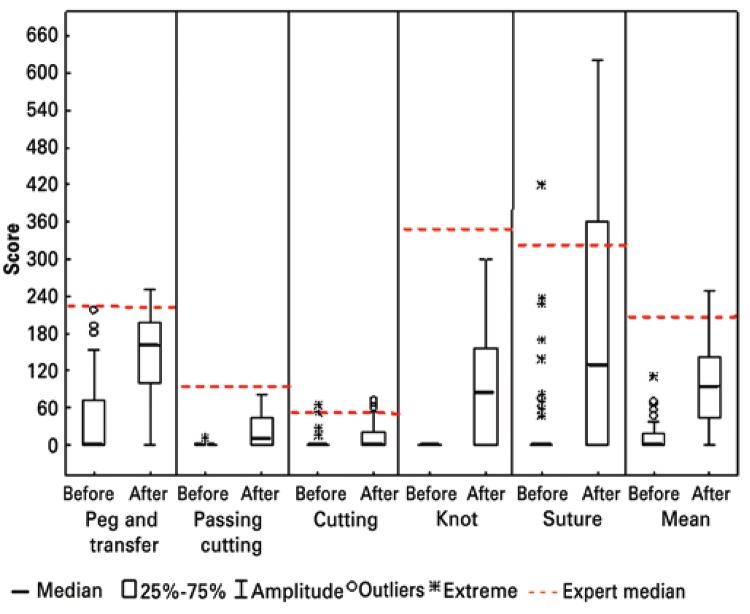
Comparing scores between Medical students and experts for different laparoscopic exercises in simulators

When comparing students to experts, training demonstrated to be an efficient form of skill acquisition, approaching the students’ scores to the experts’ average scores. In some exercises, especially the peg transfer, the students made a difference of more than 100 points (40%) as compared to their average score (Figure 3).

The univariate analysis identified that being a second-year student was a favorable factor for skill gain. In the multivariate analysis after training, this was the only variable significantly associated with improvement in skill acquisition. (Table 2).

**Table 2:** Determining factors in the acquisition of skills for laparoscopy by Medical students

Determining factors	Univariate	Multivariate		
	p value	95% CI	OR	p value
Age	0.87			
Gender	0.29			
2nd year undergraduates	0.006	1.5-14.9	4.8	0.007
Left hand dominant	0.31			
No intention in surgical carrier	0.99			
No perception of developed manual skills	0.18			0.79
No videogame practice	0.43			

95% CI: 95% confidence interval; OR: odds ratio.

## DISCUSSION

The acquisition of laparoscopic surgical skills demands specific training, preferably in surgical simulators. To maximize learning in this technique, it is important to understand how people naive to surgical exposure develop such skills. The objective of this study was assess the acquisition of laparoscopic surgical skills in Medical students trained in a simulator.

Qualification of the skills acquisition was done by performing exercises previously validated from FLS. The exercises were selected for being easily reproducible and for having been validated by other research.^(^5^,^6^,^14^,^15^)^ Only the guide-wire exercise was slightly modified in comparison to the literature, with the objective of rendering it more difficult.^(^10^)^ Each exercise had a difficulty level, the peg transfer and cutting being considered easy by the students, while passing the guide-wire, intracorporeal knot and suture were considered difficult.^(^5^)^

For each exercise, the variable used was the score, a comparative rate between the time achieved by the students and the mean time of the experts. This correction is a way to make the time obtained by the students in performing activities closer to reality.^(^6^)^ To evaluate the performance in accomplishing the tasks, both quickness and movement precision were important.^(^13^)^ In this regard, applying the penalties is key so that not only agility and speed were analyzed, but also the precision to obtain an adequate time.^(^13^)^ The penalties provided the error data obtained by the students. The average error decreased about 30% after training in the simulators.

The most important finding in this study was that there is a significant skill acquisition in Medical students who were never exposed to the practice of laparoscopy, when compared to surgeons who were instructors in laparoscopy. The students were chosen for being a population never exposed to the discipline of surgical technique and video-assisted surgery, hence rendering reliable information on skills acquisition. This was a counterpoint to the literature, since most studies are conducted with residents in surgery, which is a population already in contact with the practice of laparoscopy.^(^3^,^13^)^ A recent study conducted in Turkey demonstrated that laparoscopy learning may also be possible in a population of adolescents who have not yet entered medical school.^(^16^)^

In this study, the performance of the students before and after training improved significantly for all exercises. Bonrath et al. have equally demonstrated that Medical students are able to develop laparoscopic skills training in simulators.^(^5^)^ The effectiveness of training in simulators may be verified in other groups of learners, such as senior Medical students and residents, in which the results are similar or even better than those of the Medical students of this study.^(^12^,^17^-^20^)^ Another important indicator was the amount of performance gain by the students when compared to the instructors, contradicting other studies that observed a percentage of students who did not progress in skills acquisition.^(^9^,^21^,^22^)^

Acquiring laparoscopic skills by Medical students may be influenced by factors such as the practice of videogame, gender, and dominant hand.^(^2^,^23^-^25^)^ In this study we found, by univariate and multivariate analysis, that none of those factors was decisive in the task performance. We emphasize that, in our sample, there was no predominance of females, usually found in Brazilian universities, what was possibly related to the male preference for surgical careers. In an interesting way, students who perceived a developed manual skill and who had the intention of following surgical career did not have better performance than their peers.

Discovering the factors that influence in skill acquisition, therefore qualifying “the best future surgeon”, would be greatly interesting for surgical education. Nevertheless, that management involves an ethical dilemma. Should one select the surgeons that would have the best chances of success or should one provide conditions for any student interested in surgery to practice and reach the same level?^(^26^)^ In this sense, our study can contribute in two ways. The first is stating that every student may benefit from specific training and significantly improve their performance, with no innate factors being determinant for skill acquisition. That contradicts a recent study according to which there is innate aptitude for the skills acquisition in laparoscopic suture.^(^27^)^ The second contribution is related to the best timing of training which, based in our results, would be more advantageous when training started during the second year of Medical school.

This study had limitations, such as inexperience of the students, the short training period, and the small sample size. We also were not able to answer questions concerning the retention of videolaparoscopic skills. It should be emphasized that the performance of students in simulators does not necessarily reflect the performance of a surgeon in surgical procedures. Future studies might be designed to answer those questions.

## CONCLUSION

Medical students never exposed to laparoscopy improved their performance in accomplishing basic laparoscopic surgery tasks after training in simulators. No factors or specific skills were identified that might influence the results, except that students from the second-year had a better performance than those in the first-year of Medical school.
