# Supplementary material for: Development of laparoscopic skills in Medical students naive to surgical training
Source: Einstein (Sao Paulo). 2014 Oct-Dec;12(4):467–72. doi: 10.1590/S1679-45082014AO3237 (PMC4879913; doi:10.1590/S1679-45082014AO3237)
Supplement: Translation

# Translation: Desenvolvimento de habilidades laparoscópicas em estudantes de Medicina sem exposição prévia a treinamento cirúrgico

**Authors:** Worens Luiz Pereira Cavalini, Christiano Marlo Paggi Claus, Daniellson Dimbarre, Antonio Moris Cury, Eduardo Aimoré Bonin, Marcelo de Paula Loureiro, Paolo Salvalaggio

**Affiliations:** 1 Universidade Positivo, Curitiba, PR, Brasil.; 2 Instituto Jacques Perissat, Curitiba, PR, Brasil.

**Keywords:** Estudantes de Medicina, Laparoscopia, Modelos educacionais, Procedimentos cirúrgicos operatórios/educação

## Abstract

**Objetivo:**

Avaliar o aprendizado de habilidades laparoscópicas básicas em estudantes de Medicina submetidos a treinamento em um simulador.

**Métodos:**

Estudantes de Medicina do primeiro e segundo ano participaram de um treinamento de exercícios de laparoscopia em simuladores. Nenhum estudante havia cursado a disciplina de técnica operatória ou teve exposição prévia a cirurgias ou treinamento cirúrgico. Os mesmos tiveram seu tempo coletado antes e após treinamento de 150 minutos. A aquisição de habilidade foi medida comparando tempo e pontuações dos alunos em relação a cirurgiões instrutores em cirurgia laparoscópica.

**Resultados:**

O trabalho teve a participação de 68 alunos, com média de idade de 20,4 anos. Houve predomínio de alunos do primeiro ano (62%). Na comparação pré e pós-treino, todos os alunos obtiveram melhora de desempenho em pontuação e tempo dos exercícios, com significância estatística (p<0,001). A pontuação apresentou variação de melhora de 294.1 a 823%. Análise univariada e multivariada identificaram que alunos do segundo ano obtiveram um maior ganho de desempenho com o treinamento.

**Conclusões:**

Estudantes de Medicina nunca expostos à técnicas cirúrgicas adquirem habilidades laparoscópicas básicas em treinamento sistematizado em simuladores. Alunos do segundo ano apresentaram melhor desempenho em relação a alunos do primeiro ano.

## INTRODUÇÃO

A laparoscopia revolucionou a cirurgia nas últimas décadas. As cirurgias passaram a ser realizadas sem a necessidade de abrir a cavidade abdominal, tendo recuperação mais rápida e menos traumática. Com o surgimento dessa nova técnica, surgiram também a necessidade e o desafio de treinar cirurgiões para adquirir as habilidades necessárias para sua prática, de forma eficiente e segura.^(^1^)^ Dentre as dificuldades para a aquisição de habilidades em laparoscopia, destacam-se a perda da sensação de profundidade, a perda da sensação tátil, o aparecimento do “efeito fulcro” (instrumentos que se movimentam em um ponto fixo à parede abdominal causando movimentos inversos paradoxais), e finalmente as alterações da coordenação mão-olho.^(^2^)^ A fim de solucionar o problema, surgiu o conceito de treinamento em simuladores, também conhecidos como “caixas pretas”.^(^3^, ^4^)^

O treinamento em simuladores visa aprimorar e transferir as habilidades adquiridas do laboratório de treinamento para a sala de cirurgia.^(^5^, ^6^)^ No intuito de estabelecer um padrão de treinamento e de aquisição de habilidades mínimo, foi criado pela Sociedade Americana de Cirurgiões Gastrointestinais e Endoscópicos (SAGES) um programa educacional intitulado Fundamentos da Cirurgia Laparoscópica (FSL- *Fundaments in Laparoscopic* *Surgery*).^(^7^)^ Esse programa é baseado em uma série de exercícios validados, que foram desenvolvidos com base em habilidades únicas para a prática de laparoscopia.^(^6^, ^8^)^ Com o uso dos FLS, a aquisição de habilidades pode, assim, ser mensurada de forma qualitativa e objetiva, com base na eficiência e precisão da execução de tarefas.^(^6^)^

Além de se pensar em aquisição de habilidade, é importante identificar como o aprendizado ocorre.^(^9^)^ O aprendizado sofre influência de vários fatores complexos, dentre eles a possível habilidade inata do cirurgião e a experiência cirúrgica prévia desse profissional.^(^9^)^ A identificação de fatores que facilitem ou dificultem a aquisição de habilidade é fundamental para minimizar a curva de aprendizagem.^(^9^)^

No entanto, não se conhece em detalhes como ocorre a aprendizagem e quais são os possíveis fatores influenciadores de aquisição de habilidade. De forma mais específica, não se sabe a fundo como indivíduos sem treinamento prévio em laparoscopia adquirem e/ou desenvolvem tais habilidades específicas, qual a velocidade e os limites da aquisição, e as influências de um treinamento sistematizado.

## OBJETIVO

Avaliar a aquisição de habilidades laparoscópicas em estudantes de Medicina submetidos a treinamento em um simulador.

## MÉTODOS

Foi realizado um estudo longitudinal prospectivo com alunos de primeiro e segundo anos do curso de Medicina da Universidade Positivo, em Curitiba (PR) de 1º de junho de 2012 até 1º de setembro de 2013.

Os alunos voluntários preencheram um questionário demográfico e assinaram um Termo de Consentimento Livre e Esclarecido liberando suas informações para o estudo. O estudo foi aprovado pelo Comitê de Ética em Pesquisa da Universidade Positivo, com o parecer 51.598. (CAAE: 05247812.6.0000.0093)

O questionário coletava dados referentes a idade, sexo, ano da graduação, intenção sobre carreira cirúrgica, qual a mão dominante e habilidade manual desenvolvida pela prática de videogame.

Os alunos foram separados em grupos de treinamento em caixa simuladora por um período total de 150 minutos. Os treinamentos foram realizados em duas sessões, separadas por um intervalo de tempo de uma semana.

A coleta de tempo e a pontuação anterior ao treinamento prático em simuladores dos alunos voluntários incluídos no estudo serviram como controle.

O desempenho nos simuladores de cirurgiões instrutores do Núcleo de Cirurgia Minimamente invasiva da Universidade Positivo ou de convidados nacionais desse núcleo serviu para formar um grupo de instrutores para coleta de tempo e pontuação. Esse grupo teve como características comuns mais de 10 anos de prática em laparoscopia avançada, com pelo menos 500 cirurgias realizadas, e reconhecimento local e nacional por colegas de especialidade. O tempo e a pontuação do grupo de instrutores foram as médias de todos esses cirurgiões. Esse tempo e essa pontuação também foram considerados como objetivo final de aquisição de habilidades pelos estudantes incluídos neste estudo.

Assim, o tempo e a pontuação dos estudantes, incluídos neste estudo, foram considerados tendo em conta o tempo e a pontuação dos cirurgiões instrutores.

### Tarefas e penalidades

As tarefas utilizadas para mensuração da aquisição de habilidade em laparoscopia mimetizam as originalmente descritas nos FLS.^(^8^,^10^,^11^)^

### Transferência de tubos ou *peg transfer*

No centro do vídeo, encontra-se uma placa com 12 pinos. De um lado, encontravam-se seis anéis em seis pinos. Devia-se levantar cada um dos seis objetos com a mão não dominante e transferir com instrumental adequado para a mão dominante, seguindo para sua colocação do lado oposto. Após a realocação das seis estruturas, devia-se retornar todas para a posição inicial.^(^8^)^ O exercício iniciava-se quando as pinças apareciam na tela e terminava com a colocação do último anel. A penalidade foi aplicada para cada objeto que caiu fora do campo de visão ou fora do alcance, sendo contabilizado o valor de 10 segundos para cada erro. O tempo limite foi 300 segundos.

### Corte

No centro do vídeo, encontrava-se uma gaze medindo 10x10cm, com um círculo de 5cm de diâmetro desenhado no centro. Devia-se cortar com uma tesoura o círculo pré-desenhado.^(^8^)^ O exercício iniciava-se quando a gaze foi tocada e terminava quando todo o círculo estava recortado. A penalidade foi aplicada em comparação com o círculo cortado pelos peritos, com tolerância a um desvio máximo de 1mm. Os círculos que excedessem essa medida teriam uma penalidade contabilizada para cada milímetro. O tempo limite foi de 300 segundos.

### Passagem de fio-guia

No centro da tela, havia uma placa apresentando várias fileiras de hastes com orifícios. Devia-se guiar o fio entre os orifícios de todas as filas, sequencialmente até o término das hastes. A tarefa iniciava-se quando o fio-guia era suspenso e terminava quando ele atravessava o último orifício.^(^10^)^ O tempo limite foi de 300 segundos A penalidade foi atribuída para cada orifício pelo qual o fio-guia não passou, contabilizando 10 segundos de penalidade.^(^12^)^

### Nó intracorpóreo

No centro da tela, existia uma estrutura com um fio cirúrgico fixado. Devia-se realizar um nó duplo e quatro nós simples no fio com porta-agulha e contra porta-agulha.^(^8^)^ O exercício iniciava-se quando os instrumentais apareceram no monitor e terminava quando o último nó fosse realizado. A penalidade foi aplicada para os nós frouxos, atribuindo o valor 10 segundos a mais para a presença da frouxidão.^(^11^,^12^)^ O tempo limite foi de 300 segundos.

### Sutura

No centro do vídeo, havia um dreno de Penrose cortado. Em cada lado do corte foram demarcados dois pontos. Os pontos deviam ser unidos realizando a sutura com uso de agulha e fio.^(^8^)^ O exercício iniciava-se quando o instrumental tornava-se visível na tela e terminava quando a sutura era concluída. A penalidade foi aplicada quando havia desvio dos pontos pré-marcados ou frouxidão dos nós. Foi contabilizado o valor de 1 segundo para cada milímetro afastado da demarcação e de 10 segundos para a frouxidão do nó.^(^11^,^12^)^ O tempo limite foi de 600 segundos.

### Penalidade e pontuação

Os tempos-base e as penalidades foram estabelecidos conforme metodologia previamente publicada.^(^5^,^6^,^8^)^ A pontuação bruta de cada tarefa foi a subtração do tempo-base do tempo de execução, com posterior decréscimo das penalidades. Dessa forma, pontuações mais altas implicam em melhor desempenho. Caso o participante não conseguisse completar o exercício e excedesse o tempo limite, era atribuído tempo zero e, por consequência, pontuação zero.^(^13^)^

A pontuação foi um percentual dos pontos brutos em relação aos pontos dos peritos, segundo a fórmula abaixo:^(^6^,^13^)^



A pontuação geral de cada aluno foi a média dos pontos de cada tarefa.

Os resultados de variáveis quantitativas foram descritos por médias, medianas, valores mínimos, valores máximos e desvios padrões. As variáveis qualitativas foram descritas por frequências e percentuais. Para a comparação das avaliações pré e pós-treinamento, em relação a variáveis quantitativas, foi considerado o teste não paramétrico de Wilcoxon. Para variáveis qualitativas dicotômicas, essa análise foi feita usando-se o teste de McNemar. Na comparação de dois grupos em relação à idade, foi considerado o teste *t* de Student para amostras independentes. Para avaliação da associação entre duas variáveis qualitativas dicotômicas, foi usado o teste exato de Fisher. A condição de normalidade das variáveis foi avaliada pelo teste de Kolmogorov-Smirnov. A análise univariada foi realizada para identificar fatores que predizem ganho de desempenho dos alunos, definido como colocação final entre as 20 melhores pontuações obtidas. A análise multivariada foi então realizada para identificar fatores que isoladamente contribuiriam para esse ganho. Valores de p<0,05 indicaram significância estatística. Os dados foram analisados com o programa computacional *Statistical Package for the Social Science* (SPSS) v.20.0.

## RESULTADOS

O estudo contou com a participação de 68 alunos com distribuição semelhante de sexo. Houve predominância de alunos do 1º ano da faculdade e com intenção futura de seguir carreira em disciplina cirúrgica. As demais informações demográficas estão contidas na tabela 1.

**Tabela 1:** Dados demográficos de estudantes de Medicina submetidos à avaliação em simulador cirúrgico laparoscópico

Dados demográficos	
Idade, média	20,4 (17 ±27)
Desvio padrão	1,8
Sexo feminino n (%)	38 (55)
1^o^ ano de faculdade, n (%)	42 (62)
Carreira cirúrgica (sim), n (%)	57 (84)
Habilidade manual (sim), n (%)	37 (54)
Prática de videogame (não), n (%)	35 (51)
Mão dominante (direita), n (%)	63 (93)

Os tempos obtidos foram analisados para verificar uma possível melhora pós-treinamento. Na figura 1, os tempos, em segundos, de execução de cada exercício, medidos no pré e pós-treinamento, apresentaram evolução em todos os exercícios. O comparativo entre as duas etapas de treinamento obteve significância estatística (p<0,001).

**Figura 1:** Tempo de execução de exercícios por estudantes de Medicina, no pré e pós-treinamento, em simulador cirúrgico laparoscópico

A conversão de tempo em pontuação está apresentada na figura 2. Houve uma variação de melhora de 294,1 a 823% dependendo do exercício realizado. Todas as pontuações apresentaram p<0,001.

**Figura 2:** Desempenho antes e depois do treinamento, para diferentes exercícios laparoscópicos, em estudantes de Medicina

Na comparação do aluno com o perito, o treinamento mostrou-se uma eficiente forma de aquisição de habilidade, possibilitando àquele aproximar-se da pontuação média destes. Em alguns exercícios, em particular o *peg* *transfer,* o aluno conseguiu tirar uma diferença de mais de 100 pontos (40%) para a pontuação média do mesmo (Figura 3).

**Figura 3:** Comparação de pontuação de estudantes de Medicina e peritos para diferentes exercícios laparoscópicos em simuladores

A análise univariada identificou alunos que cursavam o segundo ano da faculdade com fator favorável ao ganho de habilidade. Na análise multivariada pós-treinamento, está foi a única variável significativamente associada com a melhoria na aquisição de habilidade (Tabela 2).

**Tabela 2:** Fatores determinantes na aquisição de habilidades de laparoscopia em estudantes de Medicina

Fatores determinantes	Univariada	Multivariada		
	Valor de p	IC95%	OR	Valor de p
Idade	0,87			
Sexo	0,29			
2^o^ ano de faculdade	0,006	1,5-14,9	4,8	0,007
Mão esquerda dominante	0,31			
Sem intenção em carreira cirúrgica	0,99			
Sem percepção de habilidade manual desenvolvida	0,18			0,79
Sem prática de videogame	0,43			
IC95%: intervalo de confiança 95%; OR: *odds ratio*.				

## DISCUSSÃO

A aquisição de habilidades em cirurgia laparoscópica requer treinamento específico, de preferência em simuladores cirúrgicos. Para maximizar o ensino dessa técnica, é necessário compreender como indivíduos sem contato e treinamento prévio adquirem e/ou desenvolvem estas habilidades. O objetivo deste estudo foi avaliar o aprendizado de gestos cirúrgicos laparoscópicos em estudantes de Medicina submetidos a treinamento em um simulador.

A forma de qualificação da aquisição de habilidades se deu pela execução de exercícios previamente validados dos FLS. Os exercícios foram escolhidos pela fácil reprodução e por terem sido validados em outras pesquisas.^(^5^, ^6^, ^14^, ^15^)^ Somente o exercício de passagem de fio-guia sofreu leves modificações em relação à literatura, com a finalidade de aumentar sua dificuldade de execução.^(^10^)^ Cada exercício apresentou um grau de dificuldade, sendo o *peg transfer* e o corte considerados pelos estudantes como de fácil execução; e exercícios considerados difíceis foram passagem de fio-guia, nó intracorpóreo e sutura.^(^5^)^

Para cada exercício, a variável utilizada foi a pontuação, uma relação comparativa entre o tempo obtido pelo aluno e o tempo médio dos instrutores. Essa correção realizada é uma forma de aproximar da realidade o tempo obtido pelos estudantes na execução das atividades.^(^6^)^ Para avaliar o desempenho na execução das tarefas, tanto a rapidez quanto a precisão dos movimentos eram importantes.^(^13^)^ Nesse quesito, é fundamental a aplicação das penalidades para que não fossem analisadas somente agilidade e velocidade, mas também a precisão para a obtenção de um tempo adequado.^(^13^)^ As penalidades forneceram os dados de erros obtidos pelos alunos. O erro médio diminuiu cerca de 30% após o treinamento nos simuladores.

O achado mais importante deste estudo foi que há uma aquisição de habilidade significativa em estudantes de Medicina que nunca foram expostos a prática da laparoscopia, quando comparados a cirurgiões instrutores em laparoscopia. Os estudantes foram escolhidos por serem uma população nunca exposta à disciplina de técnica operatória e videocirurgia, podendo oferecer dados fidedignos sobre aquisição de habilidade. Esse foi um contraponto em relação a literatura, pois a maioria dos estudos são conduzidos com médicos residentes de cirurgia, ou seja, com uma população já em contato com a prática da laparoscopia.^(^3^,^13^)^ Estudo recente da Turquia demonstrou que o aprendizado de laparoscopia também pode ser obtido em uma população de adolescentes que ainda não ingressou no curso médico.^(^16^)^

No presente estudo, o desempenho dos alunos pré e pós-treino mostrou-se significativamente melhor para todos os exercícios. Bonrath et al. igualmente demonstraram que estudantes de Medicina conseguem desenvolver habilidades laparoscópicas treinando em simuladores.^(^5^)^ A efetividade do treinamento nos simuladores pode ser comprovada em outros grupos de aprendizes, como estudantes de Medicina seniores e médicos residentes, nos quais os resultados se aproximam ou até superam os alunos de Medicina do presente estudo.^(^12^,^17^^-^^20^)^ Outro indicador importante foi a quantidade de ganho de desempenho dos estudantes em relação aos instrutores, contrapondo outros estudos que observaram um percentual de estudantes que não progrediu com relação à aquisição de habilidades.^(^9^, ^21^, ^22^)^

A aquisição de habilidades de laparoscopia por estudantes de Medicina pode ser influenciada por fatores como prática de videogame, sexo e mão dominante.^(^2^,^23^^-^^25^)^ No presente estudo, verificamos, por análise univariada e multivariada, que nenhum desses fatores foi decisivo no desempenho das tarefas. Ressaltamos que, em nossa amostra, não houve predomínio do gênero feminino, habitualmente encontrado nas universidades brasileiras, o que possivelmente esteve relacionado à preferência do gênero masculino pela carreira cirúrgica. De forma interessante, alunos que percebiam uma habilidade manual desenvolvida e com intenção de carreira cirúrgica não obtiveram desempenho superior ao de seus pares.

Descobrir os fatores que influenciem na aquisição de habilidade, qualificando, assim, o “melhor futuro cirurgião”, seria de grande interesse para a educação cirúrgica. Entretanto, essa conduta esbarra em um dilema ético. Devem-se selecionar os cirurgiões que teriam as melhores chances de sucesso ou dar condições de qualquer aluno com interesse em cirurgia de praticar e atingir o mesmo patamar?^(^26^)^ Nesse sentido, nosso estudo pode contribuir de duas formas. A primeira é postulando que todos alunos podem se beneficiar de treinamento específico e melhorar significativamente seu desempenho, não existindo fatores natos determinantes para aquisição de habilidades. Isso contraria um estudo recente, segundo o qual existe uma aptidão nata para a aquisição de habilidades em sutura laparoscópica.^(^27^)^ A segunda contribuição diz respeito a melhor época de treinamento, que, baseado nos nossos resultados, seria mais vantajoso quando iniciado a partir do segundo ano do ensino médico.

Esse estudo apresentou limitações, como a inexperiência dos estudantes, o tempo de treinamento curto e a amostra limitada. Também não conseguimos responder perguntas relativas à retenção de habilidade videolaparoscópica. Ressaltamos que o desempenho de alunos em simuladores não necessariamente reflete o desempenho de um cirurgião realizando procedimentos cirúrgicos. Futuros estudos podem ser direcionados para responder essas questões.

## CONCLUSÃO

Estudantes de Medicina nunca expostos à laparoscopia apresentaram melhora do desempenho na realização de tarefas básicas de cirurgia laparoscópica mediante o treinamento em simuladores. Não foram identificados fatores e nem habilidades específicas dos estudantes que pudessem influenciar os resultados, exceto que alunos de segundo ano obtiveram melhor desempenho que alunos do primeiro ano do curso de Medicina.
